# Transcriptomic insights into shared responses to Fusarium crown rot infection and drought stresses in bread wheat (*Triticum aestivum* L.)

**DOI:** 10.1007/s00122-023-04537-1

**Published:** 2024-01-29

**Authors:** Zhouyang Su, Shang Gao, Zhi Zheng, Jiri Stiller, Shuwen Hu, Meredith Diane McNeil, Sergey Shabala, Meixue Zhou, Chunji Liu

**Affiliations:** 1 CSIRO Agriculture and Food, St Lucia, QLD 4067 Australia; 2 Tasmanian Institute of Agriculture, University of Tasmania, Prospect, TAS 7250 Australia; 3 School of Biological Sciences, University of Western Australia, Crawley, WA 6009 Australia; 4 International Research Centre for Environmental Membrane Biology, Foshan University, Foshan, 5280 Guangdong China

## Abstract

**Key message:**

Shared changes in transcriptomes caused by Fusarium crown rot infection and drought stress were investigated based on a single pair of near-isogenic lines developed for a major locus conferring tolerance to both stresses.

**Abstract:**

Fusarium crown rot (FCR) is a devastating disease in many areas of cereal production worldwide. It is well-known that drought stress enhances FCR severity but possible molecular relationship between these two stresses remains unclear. To investigate their relationships, we generated several pairs of near isogenic lines (NILs) targeting a locus conferring FCR resistance on chromosome 2D in bread wheat. One pair of these NILs showing significant differences between the two isolines for both FCR resistance and drought tolerance was used to investigate transcriptomic changes in responsive to these two stresses. Our results showed that the two isolines likely deployed different strategies in dealing with the stresses, and significant differences in expressed gene networks exist between the two time points of drought stresses evaluated in this study. Nevertheless, results from analysing Gene Ontology terms and transcription factors revealed that similar regulatory frameworks were activated in coping with these two stresses. Based on the position of the targeted locus, changes in expression following FCR infection and drought stresses, and the presence of non-synonymous variants between the two isolines, several candidate genes conferring resistance or tolerance to these two types of stresses were identified. The NILs generated, the large number of DEGs with single-nucleotide polymorphisms detected between the two isolines, and the candidate genes identified would be invaluable in fine mapping and cloning the gene(s) underlying the targeted locus.

**Supplementary Information:**

The online version contains supplementary material available at 10.1007/s00122-023-04537-1.

## Introduction

Crop production faces constant challenge from biotic and abiotic stresses. Drought causes the most loss in crop production (FAO 2021), costing agriculture around US$80 Bln in loss opportunities worldwide (Razzaq et al. 2021). It can affect any stage in plant establishment and development including seed germination, emergence, vegetative growth, and crop yield (Ilyas et al. 2021). Drought frequency and affected areas have increased significantly during the twenty-first century (Zhao and Dai 2017). To meet the increased demands on food and feed worldwide, enhancing drought tolerance of major crop species is an urgent task for biologists and breeders alike. In addition to its direct impact on crop production, drought may also alter tolerance and resistance to a wide range of biotic and abiotic stresses (Sallam et al. 2019; Su et al. 2022). Fusarium crown rot (FCR) is amongst those biotic stresses which can be significantly affected by drought stress.

The most prevalent *Fusarium* pathogen causing FCR is *F. pseudograminearum,* although the disease can also be caused by other *Fusarium* species (Akinsanmi et al. 2004). Seedling infection is characterised by brown lesions in the crown region. Progression of the disease is characterised by increased degree of stem discolouration and spread up the inside of the leaf sheaths and stems. Severe FCR infection often leads to seedling and plant death. Whiteheads are the pronounced visual impact of severe FCR infection especially when moisture stress occurs after anthesis, and they possess shrivelled or even no grains (Chakraborty et al. 2006; Murray and Brennan 2009). Previous studies showed that, once entering plants, *Fusarium* pathogens proliferated more rapidly under drought conditions (Liu and Liu 2016), and wheat lines exhibited greater drought tolerance may trigger a stronger defence against FCR infection potentially due to lower levels of drought stress (Su et al. 2021).

Changes in transcriptome due to biotic and abiotic stresses have been investigated in many crop species. Early studies were mainly based on the use of a single genotype only, with changes in transcriptome between treated and non-treated samples being compared (Liu et al. 2015b; Zou et al. 2020). In recent years, many of such studies were based on two genotypes with contrasting differences for a targeted trait (Benaouda et al. 2023; Li and Guo 2023). Results from the use of such a pair of genotypes represent a significant improvement compared with those obtained from the use of a single genotype. However, in addition to the targeted trait, the two genotypes used in these studies likely also differ in many other aspects due to their different genetic make-ups. Thus, significant proportions of changes in gene expression detected from such studies are likely not due to the difference in the targeted trait but differences in non-targeted traits between the two genotypes.

Compared with the use of a single or a couple of genetically non-related genotypes with contrasting responses to biotic or abiotic stresses, near isogenic lines (NILs) are more effective in minimising effects caused by differences in genotypic backgrounds in transcriptomic studies, as the main difference between the two isolines for a given NIL pair is the targeted locus or gene. Therefore, NILs have been used extensively in investigating effects of specific loci for various plant morphologies (Chen et al. 2012, 2021) and disease resistance (Voss et al. 2008; Yan et al. 2011). NILs have also been used to investigate changes in transcriptomes in response to disease infections (Ma et al. 2012; Habib et al. 2016; Gao et al. 2019a; Su et al. 2021). In the study reported here, we investigated, for the first time, shared changes in the transcriptome in response to both drought stress and FCR infection based on a pair of NILs differing in both these characteristics between the two isolines.

## Materials and methods

### Creation of NILs for the FCR resistant locus Qcrs.cpi-2D

A population of F4 lines from the cross of ‘EGA Wylie/Sumai3’ was used to develop NILs targeting the locus *Qcrs.cpi-2D* (Zheng et al. 2014) based on the method of heterogeneous inbred family (HIF) (Tuinstra et al. 1997) in combination with the fast-generation technique (Zheng et al. 2013). Plants were grown in a controlled environment facility (CEF) at the Saint Lucia site of CSIRO in Brisbane. Conditions of the CEF were set as: day/night temperature 25/16 (± 1) °C, day/night relative humidity 65/80% (± 5%) and photoperiod 14 h with 500 mol m^−2^ s^−1^ photon flux density at the level of the plant canopy. An SSR marker located near the peak of the QTL (primers: forward 5′- GATAGATCAATGTGGGCCGT -3′ and reverse 5′- AACTGTTCTGCCATCTGAGC -3′) (Zheng et al. 2017) was used to identify plants heterozygous at the targeted locus. Four heterozygous plants were identified from the population. These heterozygous plants were self-pollinated and ten seeds produced from each of the plants were randomly selected for the next round of selecting heterozygous plants and self-pollination. Two homozygous plants with alternative alleles were selected at F_8_ generation for each of the four original heterozygous plants and they were treated as putative NIL pairs.

### Experiments of FCR infection and identification of true NIL pairs

A highly aggressive isolate of *F. pseudograminearum*, CS3096, collected in northern New South Wales Australia and maintained in CSIRO, was used for FCR infection. Three independent experiments were conducted against each of the putative NIL pairs, and each experiment had two replicates with 14 seedlings per isoline in each replicate. Inoculation was conducted in the CEF at St Lucia, Brisbane. Inoculum preparation and inoculation protocol were based on the method descripted by Li et al. (2008). In brief, *Fusarium* inoculum was cultured on ½ strength potato dextrose agar plates at room temperature for 4 weeks. Spores were then collected from the petri dishes and used to prepare spore suspension with double distilled water. The concentration of spore solution was adjusted to 1 × 10^6^ spores ml^−1^. Spore suspensions were stored in − 20 °C freezer and Tween 20 (0.1% v/v) was added before use.

Seeds for each isoline were sterilized by soaking in 2% hypochlorite for 5 min and then in 70% ethanol for 1 min. The sterilized seeds were rinsed three times in distilled water and placed on three layers of moist paper in petri dishes to germinate. Newly germinated seedlings (with coleoptile lengths ranging from 0.3 to 0.5 cm) were inoculated by immersing in spore suspension (water for mock-inoculation) for 1 min. The seedlings were then planted into 56 cell-kwik trays (Garden City Plastics, Australia) using the University of California potting mix (50% sand and 50% peat v/v). The trays were placed in CEF rooms with the same settings as those used for NIL development as described above.

FCR severity for each plant was scored with a 0–5 scale, where 0 represents a symptomless plant, 5 for a dead plant, and scores from 1 to 4 representing plants with incrementally increased stem browning. Scoring was carried out at 4 weeks post inoculation. A disease index (DI) was then calculated for each line using the formula of DI [sum (class frequency × score of rating class)]/[(total number of plants) × (maximal disease index)] × 100.

The NIL pair with the most significant difference in FCR severity between the two isolines were selected for this study. Samples for transcriptome analyses were obtained from two experiments. Shoot bases (1.0 cm above ground) were sampled on the fifth day after inoculation, and seven seedlings from each of the replicates were pooled together for each isoline. A total of 8 samples (2 isolines × 2 conditions (control and FCR infected) × 2 replicates) were obtained from the FCR experiments and the samples were stored at − 80 °C until use.

### Experiments of drought stress

Seeds of the NIL pairs were germinated on three-layer paper towels saturated with water in Petri dishes. The Petri dishes were placed in a 4 °C cold room for 4 days and then transferred to the work bench at room temperature for 2 days. Seedlings (coleoptiles reached about 1 cm in length) were planted individually into a pot containing 1200 g of UQ23 potting mix (pH 5.5–6.5, 70% Composted Pine Bark 0–5 mm, 30% Coco Peat, Osmocote Exact Standard 3–4 Months (~ 4 kg/m^3^) and Suscon Maxi insecticide (~ 830 g/m^3^)). The day when seedlings were planted in pots was designated as 1 DAP (days after planting). The pots were placed in a glasshouse at Saint Lucia of CSIRO, Brisbane. Settings of the glasshouse were: 24/15 (± 1) °C Day/night temperature, 50%/70% (± 5%) day/night humidity and 18 h-photoperiod.

As many studies on drought tolerance in wheat are focused on the tillering stage (Abid et al. 2016; Blum et al. 1990; Yang et al. 2021), we decided to initiate the treatment of drought stress when the first tillers become visible (about 19 DAP). At the early stages of the experiments, plants in both the control and treatment groups were well-watered every 3–4 days by soaking the pots in a large container filled with water for an hour. For the group of drought treatment, the last watering was applied on 19 DAP. For the controls, pots were watered continuously. On the same day of imposing drought stress, 300 g clay balls were added on top of each pot (both the control and treatment groups) to reduce evaporation. Water content was measured for the treatment group when leaf tissues were collected. Gravimetric soil water content (*θg*) was calculated using the formula *θg* = (wet weight-dry weight)/dry weight.

Samples for transcriptome analyses were obtained at two different time points, one at 26 DAP when no symptoms of water stress were observed from the plants of the drought treatment group, and the other at 32 DAP when symptoms of drought stress between the two isolines became obvious. Before the samples for transcriptome were taken, drought severity for each isoline was assessed based on two criteria at 32 DAP. One was the number of yellow leaves, using a scale from 0 to 10, where 0 represented a symptomless plant, and scores from 1 to10 indicated plants with an increasing number of yellow leaves. The other criterion was wilting severity of the plant, using a scale from 0 (no symptom) to 5 (severe wilting). Two independent experiments, each with two replicates, were carried out for each of these two time points. Green and fully expanded leaves near the shoot base were collected from each of the plants for RNA extraction. Leaf tissues from each of the replicates were pooled together as one sample. A total of 16 samples (2 isolines × 2 conditions [(controls and drought treatments × 2 time points (at 26 and 32 DAP, respectively) × 2 experiments)] were collected from the two drought experiments.

### RNA extraction

RNA was extracted using the PureLink™ Plant RNA Reagent (ThermoFisher). To reduce DNA contamination, RNase-Free DNase (QIAGEN) was utilized for RNA purification. The quality of the extracted RNA samples was checked using a Nanodrop-1000 Spectrophotometer and was further assessed using 1% agarose gel electrophoresis. RNA sequencing was based on the Illumina NovaSeq platform and carried out by the Australian Genome Research Facility Ltd. For each RNA sample, two technical replicates were run to ensure the reliability and reproducibility of results. NGS reads generated in the study have been deposited in NCBI under the accession number PRJNA1014793.

#### Data analysis

##### Transcriptome analysis

Raw data consisted of paired-end reads with a length of 150 bp. Quality control was performed using FastQC version 0.11.9 (Andrews 2010), followed by adapter removal and trimming of low-quality reads using Trimmomatic version 0.39 (Bolger et al. 2014). The RNA reads were then aligned to the reference genome assembly of *Triticum aestivum cv*. Chinese Spring (IWGSC RefSeq v2.1) (Zhu et al. 2021) using the multi-sample 2-pass mode of STAR version 2.7.9a (Dobin et al. 2013). Genomic feature mapping was performed using featureCounts (Liao et al. 2014). Differentially expressed genes (DEGs) were analysed using edgeR (Robinson et al. 2010) with log_2_(fold change) > 1 or < − 1 and FDR < 0.05 as thresholds. Nine pairwise comparisons were conducted to analyse the DEGs between the two isolines ‘NILR’ (hereinafter referred to as R) and ‘NILS’ (hereinafter referred to as S) under FCR infection and drought treatments. The comparisons included R^M^ versus R^I^, S^M^ versus S^I^, R^C^ versus R^D^26, S^C^ versus S^D^ 26, R^C^ versus R^D^32, S^C^ versus S^D^ 32, R^I^ versus R^D^26, S^I^ versus S^D^26, R^I^ versus R^D^32 and S^I^ versus S^D^32. Here, ‘M’ represented mock inoculation, ‘I’ represented *Fp* inoculation, ‘C’ represented mock treatment (control), ‘D’ represented drought treatment, and ‘26’ and ‘32’ represented samples obtained at 26 DAP and 32 DAP, respectively. Pairwise comparisons between drought treatment and mock treatment were conducted for each isoline and similar comparisons were also carried out to detect DEGs responsive to both FCR infection and drought stresses.

##### Detection of single nucleotide polymorphism

The raw data of RNA-seq were quality checked, and the sequence adapter was removed using FastQC version 0.11.9 (Andrews 2010) and Trimmomatic version 0.39 (Bolger 2014). Reads from the two replicates of each experiment were merged into a single file and aligned against the reference genome using the kalign function of the ngskit4b tool suite version 220,907 (available at https://github.com/kit4b). SAM files were then converted to BAM files using Samtools version 1.16.1 (Danecek et al. 2021). Variant calls were carried out using the bcftools version 1.15.1 mpileup pipeline (Danecek et al. 2021). SNPs which can cause non-synonymous variation between the R and S isolines were annotated and predicted using SnpEff version 5.1 (Cingolani et al. 2012).

### Functional enrichment analysis

A web-based resource, TriticeaeGeneTribe was employed to perform Gene Ontology (GO) term analysis (Chen et al. 2020). DEGs with logFC > 1 or logFC < -1 identified from both FCR infection and drought stress treatments were submitted to the server for annotation with following parameters: assembly: *Triticum aestivum* (IWGSC RefSeqv2.1), significance level: 0.01, multi-test adjustment method: Benjamini-Hochberg (BH), min size of genes in background set: 5 and max size of genes in background set: 1200. Transcription factor (TF) genes were identified from the DEGs identified in this study, and TF families were identified based on the database Plant TFDB version 4.0 (Jin et al. 2017).

### Identification of candidate genes underlying FCR infection and drought stress at the targeted locus

To identify candidate genes conferring FCR resistance and drought tolerance at the targeted locus of *Qcrs.cpi-2D*, several parameters were considered. They include the genetical position of the QTL which was retrieved from the QTL mapping study (Zheng et al. 2014), physical location of the locus deducted from the distribution of SNP-containing DEGs, gene expressions before and after disease infection and drought stresses, and the existence of non-synonymous variants between the R and S isolines.

## Results

### Phenotypic responses of the NILs to FCR infection and drought stresses

**Fig. 1:**
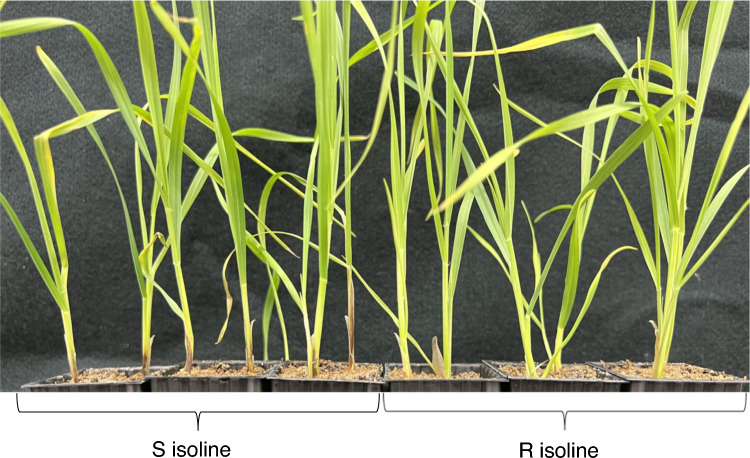
Differences in Fusarium crown rot severity (stem or seedling base browning) between the S and R isolines 3 weeks after inoculation

Four weeks following FCR inoculation, differences in disease symptom between the R and S isolines were assessed. Browning lesions at the seedling bases of the S isolines were clearly visible but not for the R isolines. Mean disease indexes between R and S for the four NIL pairs ranged from 20% to 42.5%. The difference in FCR severity between the S and R isolines for the NIL pair selected for this study became clear at about 3 weeks following infection (Fig. 1).

**Fig. 2:**
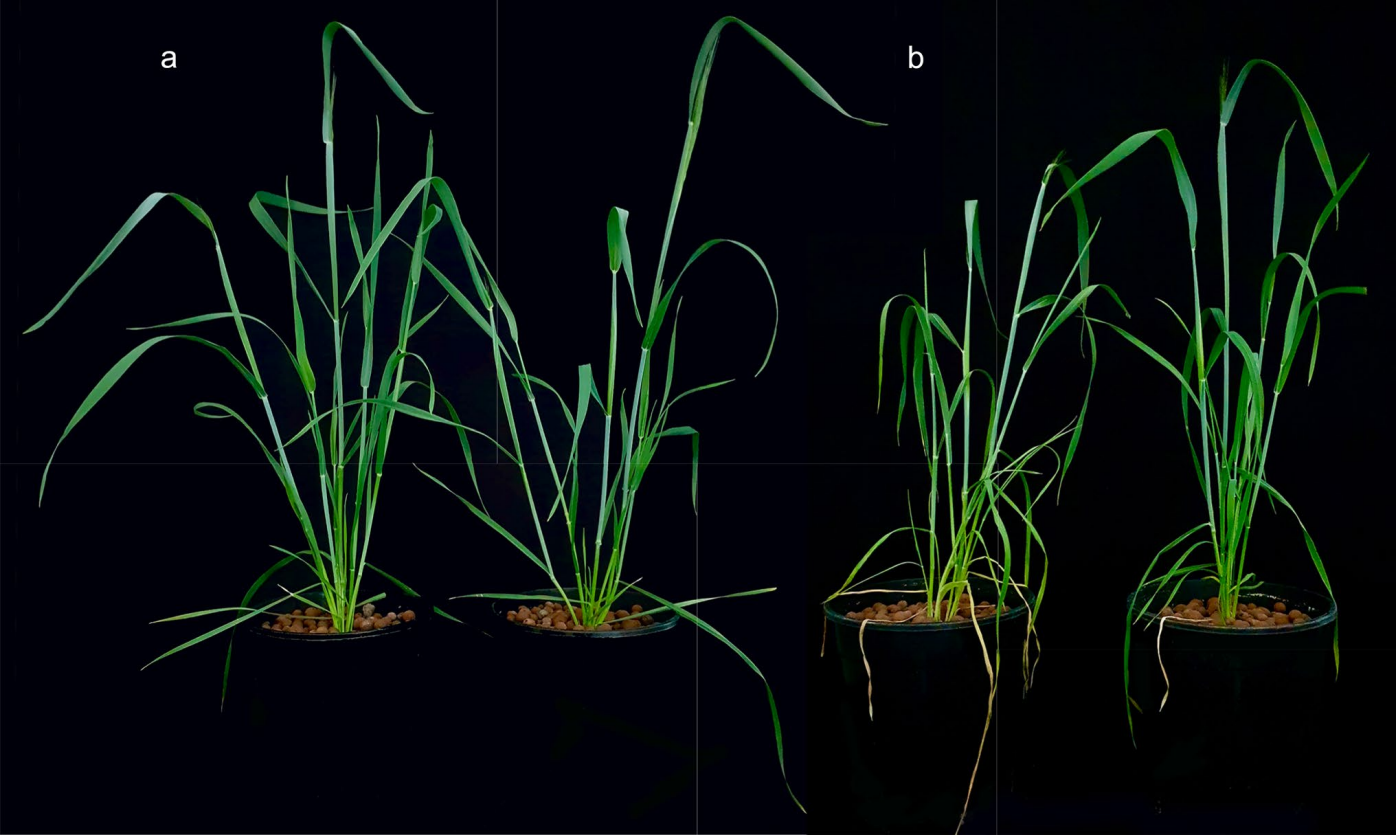
Differences in severity of drought stress between the R and S isolines **a** under well-watered condition (S line on left), and **b** under drought stress (S line on left) at 34 days after planting#. # Severities of drought stress shown by the two isolines were measured using the number of yellow leaves (average 2 for the R isoline and 7 for the S isoline) and levels of leaf wilting (1 for the R isoline and 3 for the S isoline)

A series of preliminary experiments were conducted to determine the optimum times to take samples for transcriptome analyses. When watering was stopped on 19 DAP, the symptom of drought stress (leaf wilting) became visible at about 32 DAP. We thus decided to take the first lot of samples at 26 DAP (which was about 1 week before the symptom of drought stress became visible) and the second lot of samples at 32 DAP. When the first lot of samples was taken at 26 DAP, mean gravimetric soil water content with the S isoline was 0.83 ± 0.01, and for those with the R isoline was 0.87 ± 0.01. When the second lot of samples was taken at 32 DAP, mean gravimetric soil water content with the S isoline was 0.20 ± 0.02, and for those with the R isoline was 0.24 ± 0.04. At 32 DAP, plants of both groups of the control and drought treatments had reached the booting stage. The two isolines had similar numbers of tillers and plant height under well-watered conditions. However, they showed clear difference in severity of drought stress (Fig. 2). The average number of yellow leaves in the R isoline was 2 and it was 7 in the S isoline. The wilting severity was scored 1 in the R isoline, whilst it was 3 in the S isoline.

### DEGs induced by FCR infection

A total of 545.90 Gb sequence data were obtained from samples obtained from experiments on both FCR and drought treatments. Reads that passed through quality tests were used to assess FCR-induced DEGs for both the R and S isolines first (Fig. S1). This assessment identified 84 DEGs from the R isoline. Of them, 79 were up-regulated and 5 down-regulated. As many as 240 DEGs were identified from the S isoline, with 205 being up-regulated and 35 down-regulated. Amongst these DEGs, 35 were shared between the two isolines. Interestingly, the 35 DEGs shared by the two isolines showed similar expressions between the two isolines, i.e. they were either up- or down-regulated in both isolines when compared with their respective controls.

### DEGs induced by drought stresses at two different time points of treatments

In analysing DEGs induced by drought stresses, pairwise comparisons between mock and drought treatments for each isoline were carried out first. At 26 DAP, 10,848 DEGs were detected in the R isoline following drought treatment (Fig. S2a). The number of up-regulated genes was roughly equivalent to the number of down-regulated ones. Similarly, a large number of DEGs (10,099) were also detected from the S isoline following drought treatment (Fig. S2a). Of them, 5,058 were up-regulated and 5,041 down-regulated. Amongst these DEGs induced by drought treatment at 26 DAP, 2,133 were found from both isolines. Of these, 36 genes were up-regulated and 14 down-regulated in the R isoline.

Compared with those detected at 26 DAP, the numbers of DEGs induced by drought treatment at 32DAP were even larger. When compared to the controls, 12,517 DEGs were identified from the R isoline (Fig. S2b), with 5,453 being up-regulated and 7,064 down-regulated. A total of 13,761 DEGs were identified from the S isoline (Fig. S2b), with 6,331 being up-regulated and 7,430 down-regulated. Comparing DEGs responsive to drought treatment at 32 DAP between the two isolines uncovered 3,256 shared ones. Of them, only 45 showed opposite expression patterns and they were all down-regulated in the R isoline and up-regulated in the S isoline following drought stress.

**Fig. 3:**
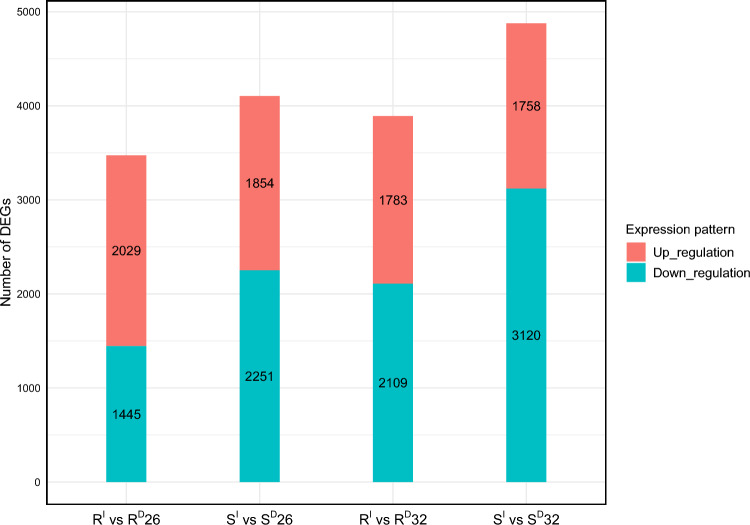
Shared genes identified between FCR infection and drought stresses Red represented up-regulated and cyan for down-regulated genes

Comparing transcriptomic changes of the R isoline in response to drought stress found that 4,565 DEGs were shared between the two time points of treatments (Fig. S2c). Amongst them, 1,245 were up-regulated at one time point but down-regulated at the other. For the S isoline, 4,298 DEGs were detected from both time points of the treatments and about a quarter of them exhibited converse expressions between the two time points (Fig. S2d). Shared DEGs induced by both FCR infection and drought stresses at 26 DAP were also identified (Fig. 3). In the R isoline, 2,029 up-regulated and 1,445 down-regulated DEGs were identified from both stress treatments. In the S isoline, 1,854 up-regulated and 2,251 down-regulated DEGs were identified. At 32 DAP, 1,783 up-regulated DEGs and 2,109 down-regulated DEGs were shared between FCR infection and drought stress. In the S isoline, 1,758 up-regulated and 3,120 down-regulated DEGs were shared between these two stresses.

### Distribution of SNP-containing DEGs in the wheat genome

**Fig. 4:**
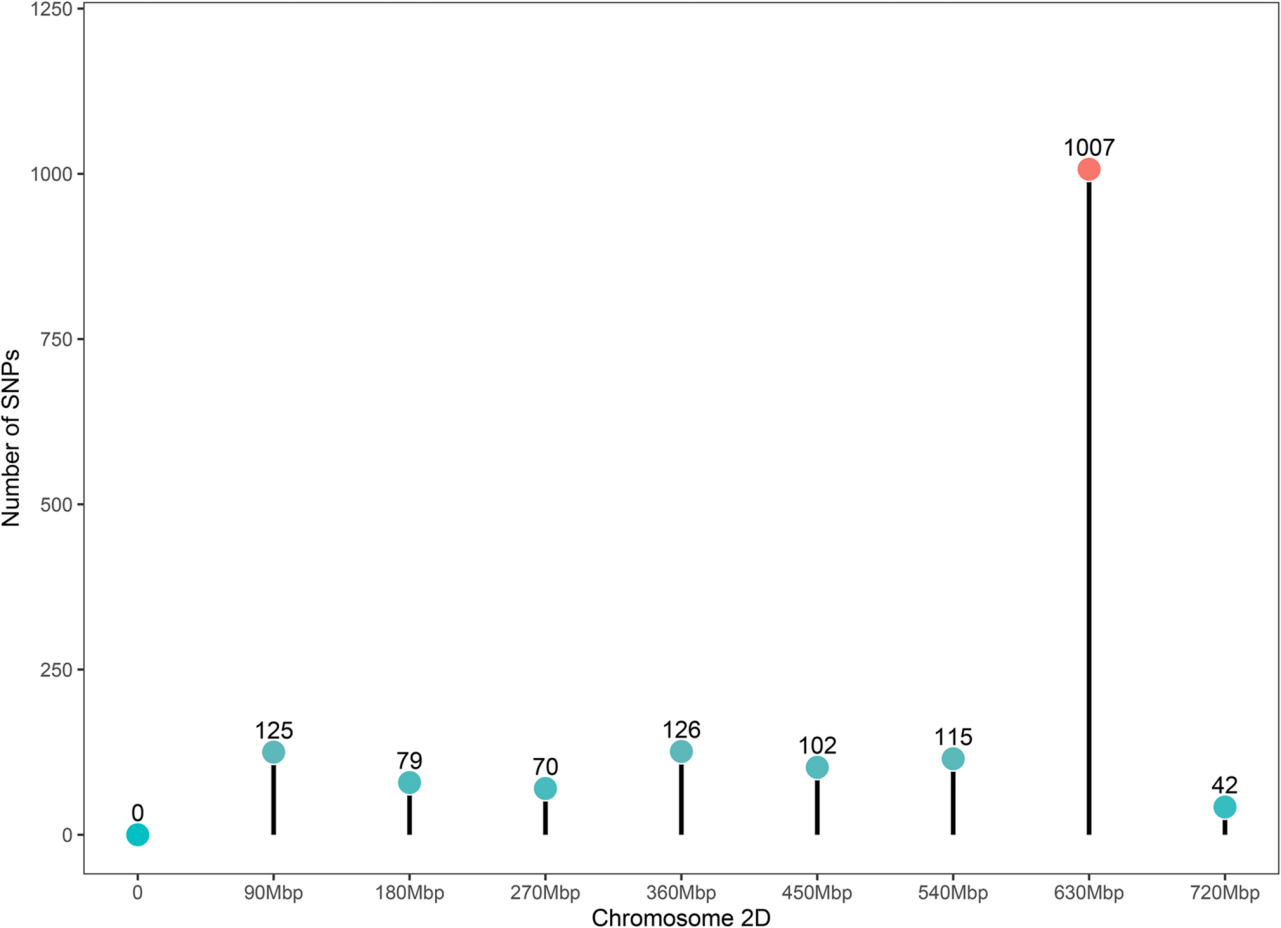
Distribution of SNP-containing DEGs between the R and S isolines on chromosome 2D. The value for the region of the targeted QTL was marked as red

Distributions of SNP-containing DEGs for the two isolines were also assessed. A total of 1,666 non-redundant homozygous SNP-containing DEGs between the R and S isolines were detected. Although these DEGs were found on each of on each of the 21 chromosomes, their distributions are highly skewed in the wheat genome. As expected from the location of the targeted QTL, a large proportion of them (about 60%) located on the distal part of chromosome arm 2DL (Fig. 4).

### Functional enrichment analysis of DEGs responsive to FCR infection and drought stress

Fourteen GO terms were identified from the R isoline, and an equal number were identified from the S isoline following FCR infection. Only two (or about 14%) of them were shared between the R and S isolines including ‘cyanate hydratase activity’ and ‘cyanate catabolic process’ (Table S1). Clearly, significant difference exists in gene networks activated by FCR infection between the two isolines.

For the drought treatment at 26 DAP, 78 GO terms were identified from the R isoline and 71 were detected from the S isoline. Thirty (or about 40%) of these GO terms were detected from both isolines. Other GO terms, including ‘chloroplast nucleoid’ and ‘photosystem II oxygen evolving complex’, were amongst the GO terms activated in the R isoline only, and ‘photosystem I’ and ‘proton-transporting ATP synthase activity’ were amongst those activated in the S isoline only.

**Fig. 5:**
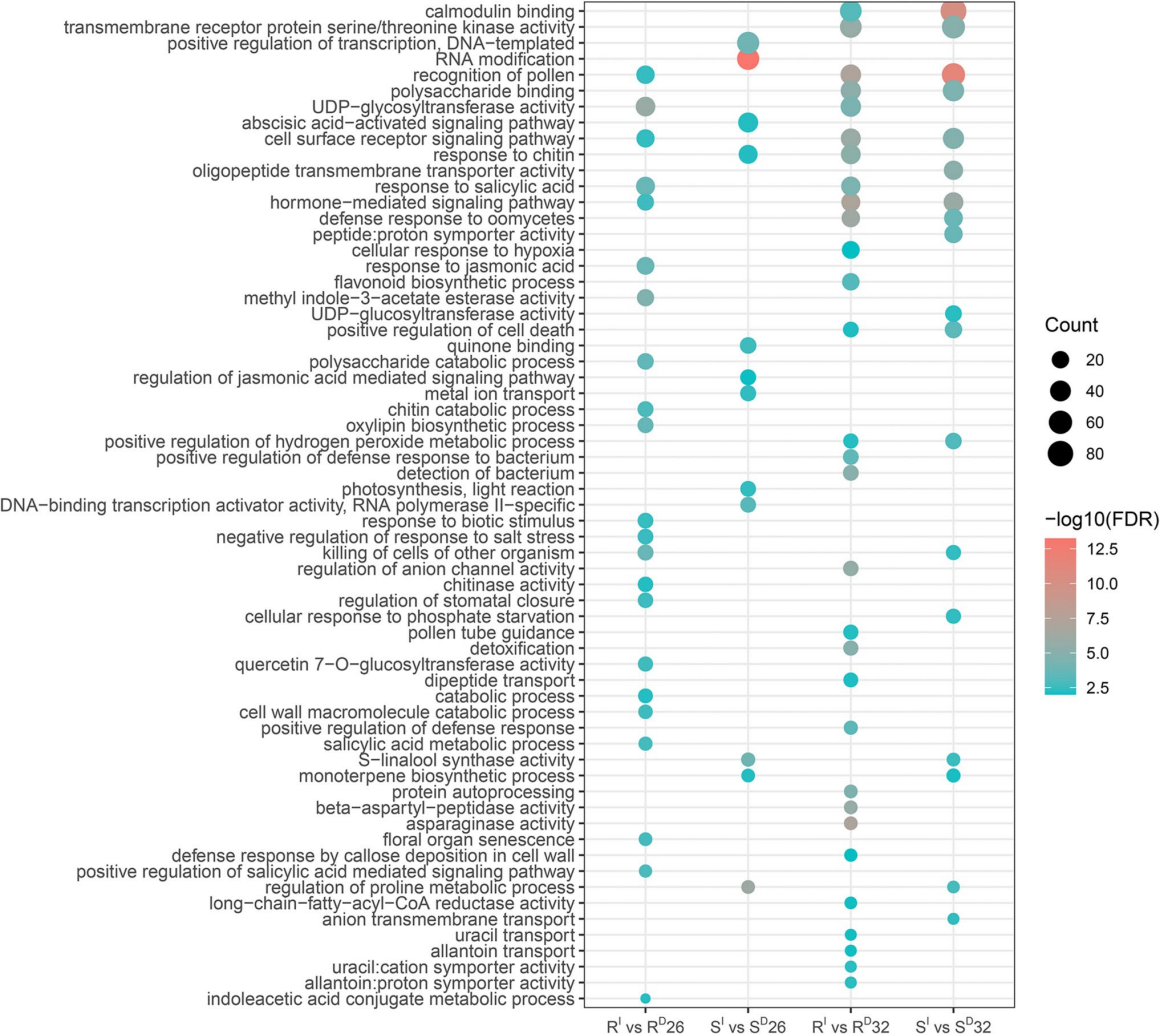
Significantly enriched GO terms shared between FCR infection and drought stresses. The bubble chart showed enriched GO terms for pairwise comparisons between FCR infection and drought stresses. Size of dots represented the number of enriched genes and colour scale showed enrichment score

Twenty GO terms were detected from both FCR infection and drought treatment at 26 DAP between the two isolines (Fig. 5, Table S2). In the R isoline, several of these GO terms were derived from up-regulated genes involved in the production of phytohormones such as ‘response to salicylic acid’ (1.94 × 10^–4^), ‘response to jasmonic acid’ (1.94 × 10^–4^) and ‘positive regulation of salicylic acid mediated signalling pathway’ (1.24 × 10^–3^). They also included two GO terms from the down-regulated genes which were involved in ‘UDP-glycosyltransferase activity’ and ‘quercetin 7-O-glucosyltransferase activity’. Of the GO terms detected from the S isoline, only one was from up-regulated genes and they were related to ‘RNA modification’ (5.75 × 10^–14^).

For the drought treatment at 32 DAP, 100 GO terms were detected from the R isoline and 109 from the S isoline. Of these GO terms, 51 (about 49%) were shared between the two isolines. Forty-nine of these GO terms were identified from the R isoline. Fifty-eight GO terms were identified from the S isoline.

Thirty shared GO terms were detected between FCR infection and drought treatment at 32 DAP between the two isolines (Fig. 5, Table S2). Eight of them were from up-regulated genes in the R isoline. They included ‘asparaginase activity’ (8.73 × 10^–8^), ‘beta-aspartyl-peptidase activity’ (1.62 × 10^–6^) and ‘long-chain-fatty-acyl-CoA reductase activity’ (8.48 × 10^–3^). Twenty-two GO terms with down-regulated genes were identified between FCR infection and drought treatment. In the S isoline, three GO terms with up-regulated genes were identified from both FCR infection and drought treatment. Fifteen GO terms with genes exhibiting down-regulation were identified from both stress treatments.

Eight GO terms induced by FCR infection and drought stresses at both 26 DAP and 32 DAP were detected from the two isolines (Fig. 5). Five of them were from the R isoline. Notably, all of them were from up-regulated genes under FCR infection and drought treatment at 26 DAP but down-regulated genes under FCR infection and drought treatment at 32 DAP. Three GO terms were detected from the S isoline, and they were all from down-regulated genes.

### Transcription factors detected under FCR infection and drought stresses

**Fig. 6:**
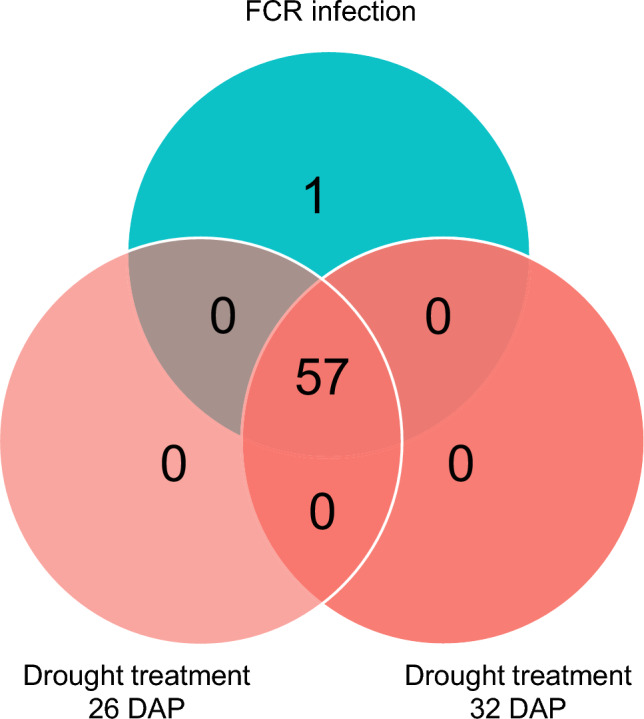
Overlapping TF families between FCR infection and drought treatment at both 26 DAP and 32 DAP

**Fig. 7:**
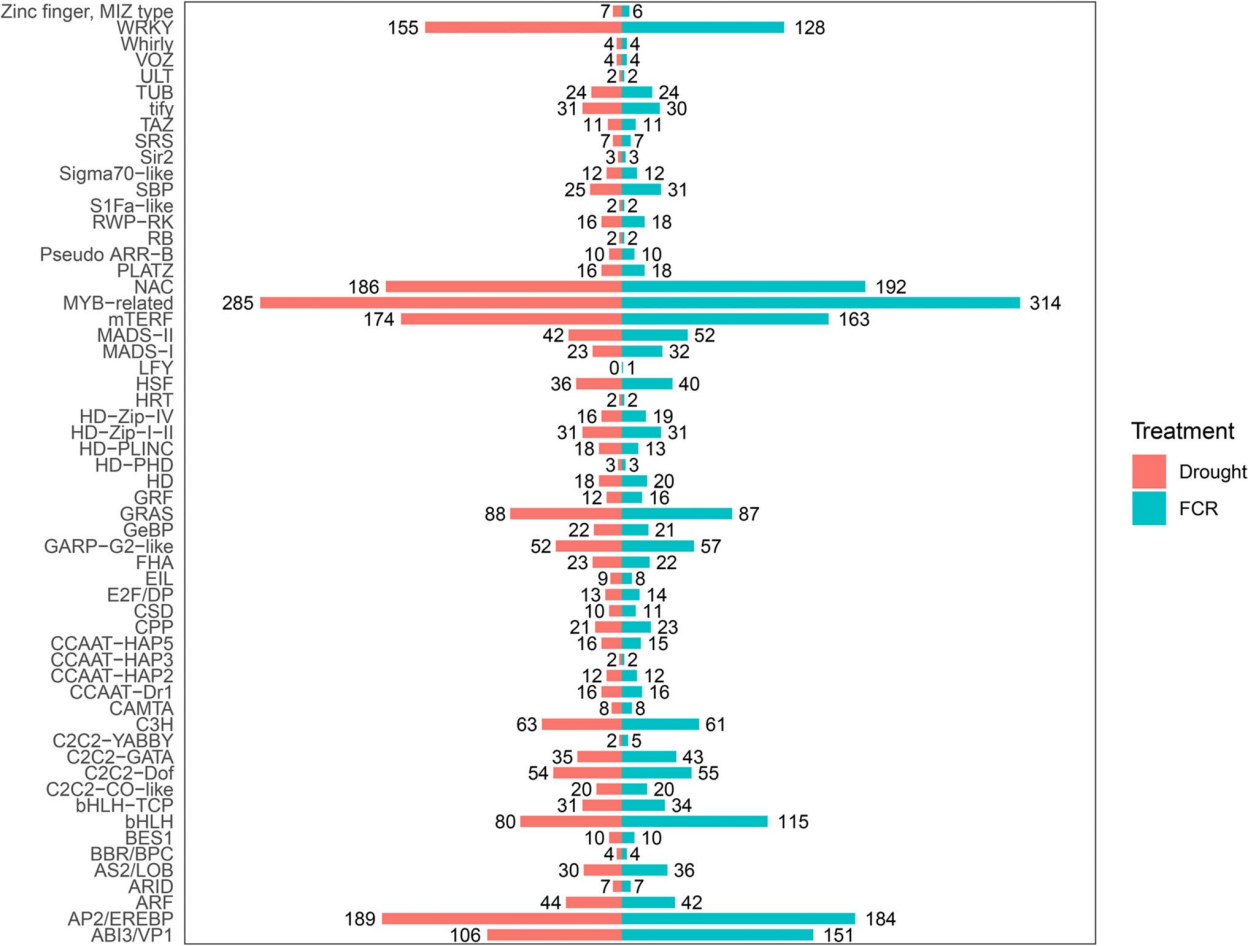
The number of TF genes and corresponding families identified under FCR infection and drought stress Results from combined drought treatments at 26 DAP and 32 DAP are shown in red, and those from FCR infection were in cyan

A total of 2,295 TF genes belonging to 58 families were identified from FCR infection between the two isolines. For drought stress at 26 DAP, 1,988 TF genes belonging to 57 families were identified. Interestingly, all but one of the TF families identified from drought stress at 26 DAP were identical to those identified from the FCR infection (Fig. 6). The exception was the LFY family which was not detected from drought stress. For drought treatment at 32 DAP, 2,144 TF genes were identified. Importantly, these genes belonged to the same 57 families as those detected for drought stress at 26 DAP (Fig. 6). Genes belonging to MYB-related TF family were found to be prominent in conferring both FCR resistance and drought tolerance (Fig. 7).

### Candidate genes conferring FCR resistance and drought tolerance at the targeted locus

**Fig. 8:**
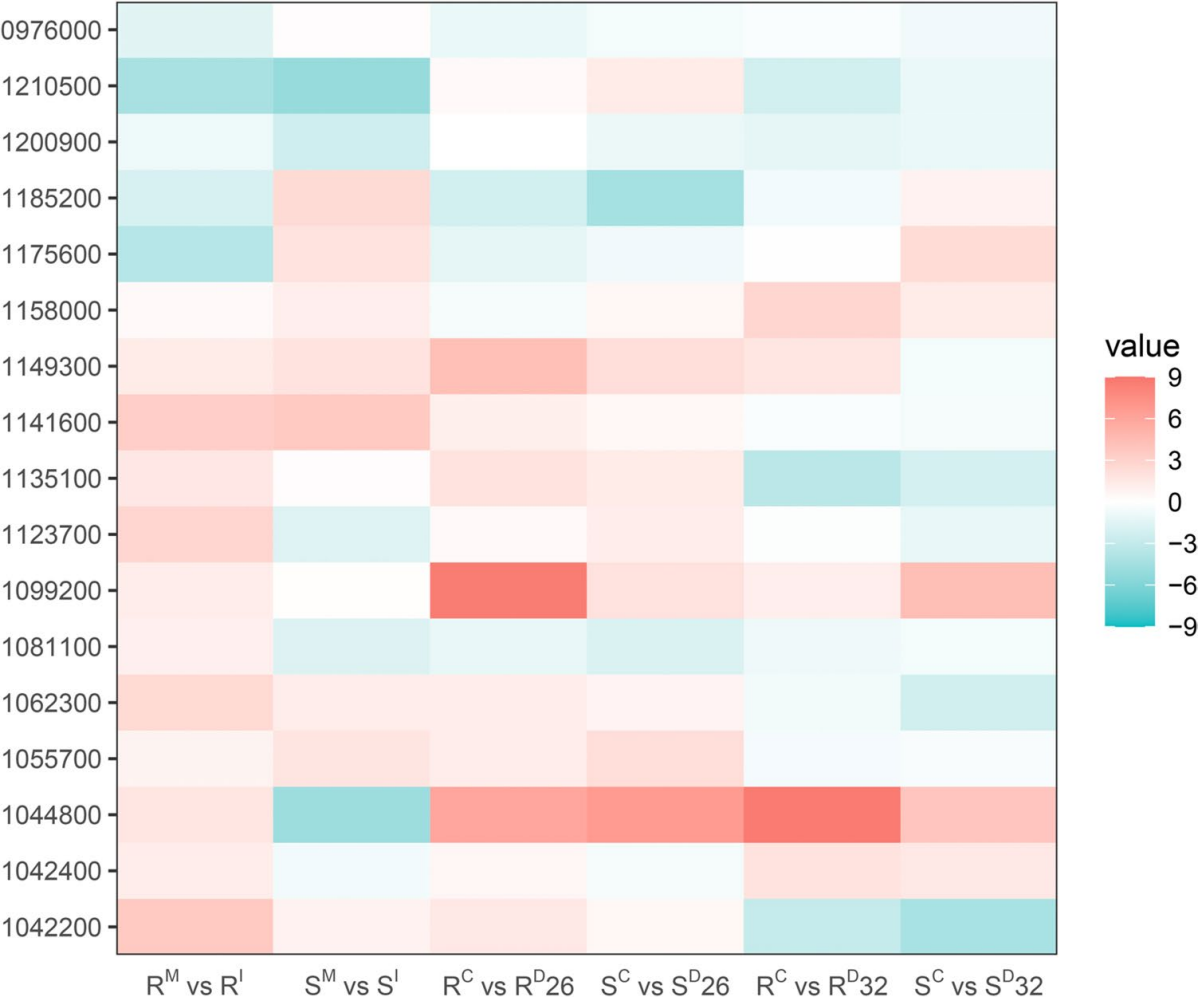
Expression of candidate genes amongst pairwise comparisons under FCR infection and drought stresses. Only partial IDs were given and the common first half of the IDs (*TraesCS2D03G*) was not included. Colour scale represented expression level, red for high expression, cyan for low expression and white for no expression

Based on the distribution of SNP-containing DEGs across chromosomes in wheat genome, the targeted locus could be positioned in the interval between 540 and 630Mbp (Fig. 4). A total of 175 DEGs induced by both FCR infection and drought stress were identified in this interval. SNPs in 21 of these genes could lead to non-synonymous variations in the coding regions between the R and S isolines. Of them, 17 genes were expressed under both FCR and drought condition stresses (Fig. 8). Several of these genes were up-regulated in the combination of FCR infection and drought stress at 26 DAP in both isolines, whereas more genes were down-regulated under the combination of FCR infection and drought stress at 32 DAP. Nine of these genes showed similar expression patterns between FCR infection and drought stresses at both 26 DAP and 32 DAP in the R isoline, and six in the S isoline.

## Discussion

The phenomenon that cereal crops suffer more yield loss from FCR infection under drought stress has been widely known. Reports on possible mechanisms underlying the phenomenon are highly limited. Aimed to understand their relationships, we obtained a pair of NILs targeting a locus conferring resistance to both FCR infection and tolerance to drought stress. Transcriptomes were obtained from both isolines following FCR infection and drought stresses at two different time-points of treatments. Results from analyses of DEGs, GO terms and TFs revealed that significant differences in response to FCR infection and drought stresses exist between the two isolines as well as between the two different time points of drought stresses. Nevertheless, changes in regulatory frameworks induced by these two types of stresses were significantly overlapped. Several candidate genes underlying the targeted locus were identified based on the physical location of the targeted locus deducted from the distribution of SNP-containing DEGs across the 21 wheat chromosomes, changes in expression associated with disease infection or drought stresses, as well as sequence variants between the two isolines.

Compared with those induced by drought stress, the numbers of DEGs induced by FCR infection in both the R and S isolines were much smaller. The difference in DEG numbers seems to reflect differences in complexities of mechanisms underlying these two different cues. Clearly, drought tolerance is more complex in compairing with that of FCR resistance. It is known that drought responsive strategies vary from changes in stomata and root structures to osmotic adjustment, antioxidative defence, photohormones, cellular membrane stability and water-use efficiency (Blum 2017; Abobatta 2019; Ilyas et al. 2021; Tardieu 2022). The interplay of these characteristics contributes to the intricate complexity of drought tolerance, setting it apart from more focused defence mechanisms observed in response to specific stressors like pathogen infection (Ghozlan et al. 2020). Although the total numbers DEGs detected under FCR infection and drought stresses are very different, TF families responsive to these two different cues are similar (Figs. 6, 7). Results from this study showed that MYB-related GO terms are over-represented in both FCR infection and drought stress. These results were consistent with those from previous reports showing that that MYB-related gene family of TFs were involved in regulating various physiological processes in plants, including drought tolerance in potato (Shin et al. 2011), salt tolerance in tobacco (Ganesan et al. 2012), and cold (Su et al. 2010), drought and salt tolerance in rice (Xiong et al. 2014).

Due to their relatively large numbers, it was not surprising that shared DEGs and GO terms were detected between FCR infection and drought stresses. However, the degree of overlapping in activated TF families between FCR infection and drought stresses was phenomenal. Of the 58 TF families detected from FCR infection, 57 were also detected from drought stresses (Fig. 6). The very high level of overlap in induced TF families between the two different stresses suggests that plants might utilize common sets of regulatory frameworks to cope with diverse environmental adversities (Manna et al. 2021). This tactic might allow plants to optimize their genetic repertoire, ensuring a broad-based response even if the specific genes differ across stresses (Atkinson and Urwin 2012). Moreover, interactions between different stress responses can amplify this unified defence mechanism even when the immediate responsive genes vary between stresses (Tsuda and Somssich 2015). The activation of the LFY family under FCR infection but not under drought stress is the only exception amongst TF families induced by the two different stresses. Traditionally tied to floral development (Moyroud et al. 2010; Chahtane et al. 2013; Yamaguchi et al. 2013), the LFY family may harbour a more expansive role than established functions. This includes a potential, yet underexplored, involvement in disease response. The disease might trigger secondary growth and developmental changes in plants, leading to enhanced LFY expression. Such indirect impacts also underscore intricate balance between plant development and defence. Pathogens might manipulate or interfere with developmental pathways to facilitate infection (Kazan and Lyons 2014; Ma and Ma 2016). Future research focused on the specific role of LFY in disease defence would be crucial for a deeper understanding of the multifaceted nature of plant stress responses.

One of the advantages in using NILs to identify candidate genes underlying a targeted locus is that such genes should possess non-synonymous variants between the two isolines. Available reports indicate that at least two of these candidate genes confer resistance or tolerance to both biotic and abiotic stresses (Table 1). One of them is *TraesCS2D03G1055700*. A homologue of this gene, *OsMB6*, showed a positive correlation with botic stresses and responded to drought and salt stress in *Arabidopsis* and rice (Kushwaha et al. 2016). The other one is *TraesCS2D03G1062300*. A homologue of this gene, *XA21*, was shown to confer broad resistance to bacterial blight resistance in rice (Jiang et al. 2013), and it also served as a mediator for drought stress and plant growth under water-deficient environment (Shamsunnaher et al. 2020). Previous reports also show that several of the candidate genes identified in this study confer tolerance to either biotic or abiotic stresses. They include *TraesCS2D03G1158000*. A homologue of this gene, *PI21*, is a well-characterised gene conferring durable and race non-specific resistance to blast disease in rice (Fukuoka et al. 2009). Another one is *TraesCS2D03G1081100*. A homologue of this gene, *BHLH112*, was related to drought tolerance in peanut (Li e al. 2021) and salt tolerance in *Arabidopsis* (Liu et al. 2015a).

**Table 1:** Candidate genes and their known functions

Candidate gene	Homologue	Species	Functional keywords	Known functions	References
TraesCS2D03G1042200	WAK2	*Arabidopsis*	Serine/threonine-protein kinase Transferase	Pectin signal relay Defence activation Fusarium head blight resistance	Kohorn et al. (2009) Zarattini et al. (2017) Gadaleta et al. (2019)
TraesCS2D03G1044800	OsRZF1	Rice	Metal-binding Zinc	Mg homeostasis maintain	Kobayashi et al. (2023)
TraesCS2D03G1055700	OsMB6	Rice	Ubl conjugation pathway	Drought tolerance Disease resistance	Kushwaha et al. (2016)
TraesCS2D03G1062300	XA21	Rice	Receptor Serine/threonine-protein kinase Plant defence	Drought mediator Immune receptor Disease resistance mediator	Jiang et al. (2013) Shamsunnaher et al. (2020)
TraesCS2D03G1081100	bHLH112	*Arabidopsis*	DNA-binding Transcription regulation	Suppression of lateral root emergence Transcriptional activator Stress tolerance Regulation of ABA signalling	Wang et al. (2013) Liu et al. (2015a) Li et al. (2021)
TraesCS2D03G0976000	RUK	*Arabidopsis*	Serine/threonine-protein kinase Transferase Cell cycle Cell division	Functions in cell plate formation, cytokinesis, and microtubule organization	Krupnova et al. (2013)
TraesCS2D03G1158000	PI21	Rice	Plant defence	Disease resistance	Fukuoka et al. (2009)
TraesCS2D03G1200900	HIPP39	*Arabidopsis*	Metal-binding	Homeostasis and detoxification	Liu et al. (2023)
TraesCS2D03G1042400		Wheat	DNA-binding ABA signalling pathway Transcription regulation	BZIP domain-containing protein (predicted)	
TraesCS2D03G1099200		Wheat	Unknown	Oxidative stress 3 (predicted)	
TraesCS2D03G1123700		Wheat	Serine/threonine-protein kinase Transferase Receptor	Protein kinase domain-containing protein (inferred from homology)	
TraesCS2D03G1141600		Wheat	Glycosyltransferase Transferase	Glycosyltransferase (predicted)	
TraesCS2D03G1149300		Wheat	Unknown	Cation-transporting P-type ATPase C-terminal domain-containing protein (predicted)	
TraesCS2D03G1175600		Wheat	Unknown	Anthocyanin 5-aromatic acyltransferase (inferred from homology)	
TraesCS2D03G1185200		Wheat	Unknown	SPRY domain-containing protein (predicted)	
TraesCS2D03G1200900		Wheat	Unknown	HMA domain-containing protein (predicted)	
TraesCS2D03G1210500		Wheat	Unknown	Amine oxidase domain-containing protein (predicted)	

Cells were left empty when the relevant information is not available

It is of note that, like those identified from any transcriptomic analysis, candidate genes identified in this study also need to be further evaluated. Quantitative polymerase chain reaction (qPCR) has been widely used in validating the expression levels of such genes (Moatamedi et al. 2023; Qalavand et al. 2023). As having been reported in many studies, data from qPCR analyses are usually consistent with expression patterns obtained from transcriptome or RNA-seq analyses (Habib et al. 2018; Gao et al. 2019a, 2023; Soheili-Moghaddam et al. 2022). It is believed that the occasional discrepancy between RNA-Seq and qRT-PCR data could be due to lack of specificity of the relevant primers leading to non-specific amplification, or detection of expression of paralogous genes (Sudheesh et al. 2016; Braich et al. 2019). The technique of qPCR is simple, and it is still widely used in gene validation. However, levels of expression alone cannot be used to determine gene functions or values. Although some of them are technically demanding and time consuming, several highly reliable methods for elucidating gene functions and values are available. They include genetic transformation (Zheng et al. 2006; Forner et al. 2023), EMS (Ethyl methanesulfonate)-induced mutagenesis (Ramirez Gaona et al. 2023), and targeted gene editing based on RNA interference (RNAi) or CRISPR (Clustered Regularly Interspaced Short Palindromic Repeats) (Gao et al. 2019b; Ramirez Gaona et al. 2023).

The NIL pair used in this study was generated using the approach of HIF (Tuinstra et al. 1997) which was based on self-pollinating of plants heterozygous at the targeted locus. Based on random recombination, high quality of NILs could be expected after seven generations of self-pollination and selection (Ma et al. 2012; Habib et al. 2016; Gao et al. 2019a). However, it is well known that, in addition to the number of generations, many other factors may also affect the rates of recombination (Gaut et al. 2007). Importantly, recombination rates are not distributed evenly across any chromosome in a genome (Liu et al. 1994; Konduri et al. 2000). Thus, as shown in this publication (Fig. 4), genetic differences between the isolines for some genomic regions can be higher than those estimations based on the cycles of self-pollination or random recombination.

The distribution of SNPs across the 21 wheat chromosomes does not only confirm the location of the targeted locus on chromosome 2D but also provide a much-refined physical interval in comparison with that achieved based on QTL mapping (Zheng et al. 2014). The SNP-containing genes within the interval can be conveniently used to develop locus-specific markers which can be used to facilitate the development of markers tightly linked to a given locus via fine mapping (Habib et al. 2018; Jiang et al. 2019; Gao et al. 2020). Clearly, the NILs generated, and candidate genes and the SNP-containing genes identified from this study should be invaluable to clarify whether the targeted locus conferring both drought tolerance, and FCR resistance is conditioned by a single gene or two or more tightly linked ones.

### Supplementary Information

Below is the link to the electronic supplementary material.

Supplementary file1 (PDF 17 kb)

Supplementary file2 (XLSX 45 kb)
