# Supplementary material for: Transcriptomic insights into shared responses to Fusarium crown rot infection and drought stresses in bread wheat (Triticum aestivum L.)
Source: Theor Appl Genet. 2024 Jan 29;137(2):34. doi: 10.1007/s00122-023-04537-1 (PMC10824894; doi:10.1007/s00122-023-04537-1)
Supplement: Supplementary file 1 — Supplementary file1 (PDF 17 kb) [file 122_2023_4537_MOESM1_ESM.pdf]

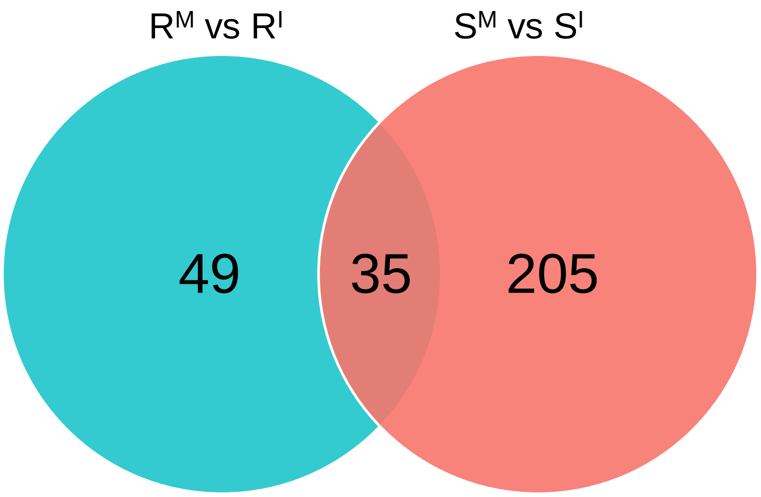

Fig. S1 DEGs induced by FCR infection between the R and S isolines

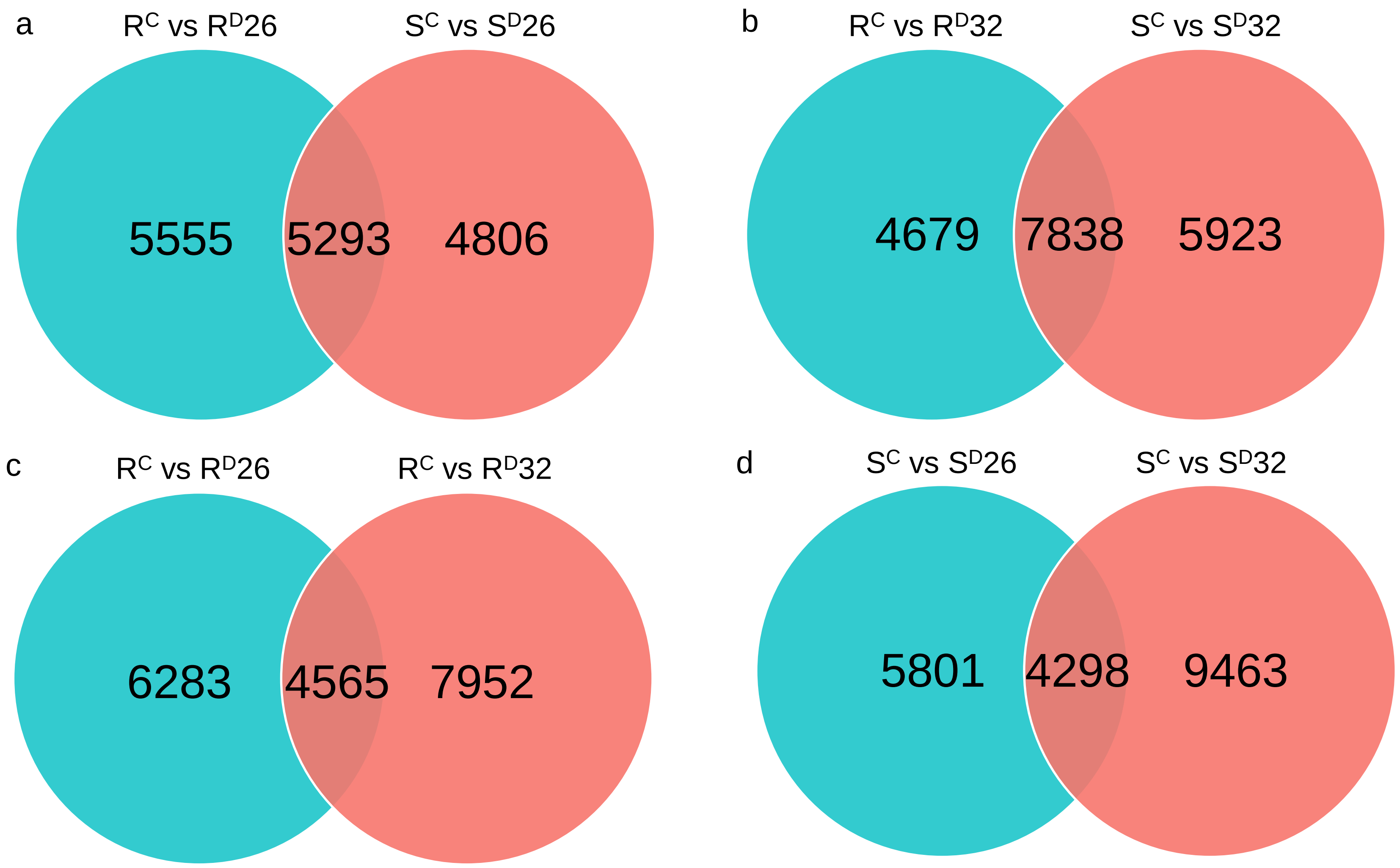

Fig. S2 DEGs induced by drought treatment at 26 DAP and 32 DAP for each isoline
